# Staying below the Radar: Unraveling a New Family of Ubiquitous “Cryptic” Non-Tailed Temperate Vibriophages and Implications for Their Bacterial Hosts

**DOI:** 10.3390/ijms24043937

**Published:** 2023-02-15

**Authors:** Panos G. Kalatzis, Jesper Juel Mauritzen, Caroline Sophie Winther-Have, Slawomir Michniewski, Andrew Millard, Maria Ioanna Tsertou, Pantelis Katharios, Mathias Middelboe

**Affiliations:** 1 Marine Biological Section, Department of Biology, University of Copenhagen, 3000 Elsinore, Denmark; 2 Department of Genetics and Genome Biology, University of Leicester, University Road, Leicester LE1 7RH, UK; 3 Institute of Marine Biology, Biotechnology and Aquaculture, Hellenic Centre for Marine Research, Former American Base of Gournes, 71500 Heraklion, Greece; 4 Department of Biology, University of Southern Denmark, 5230 Odense, Denmark

**Keywords:** non-tailed phages, NO16, *Vibrio*, ubiquitous presence, integration, *dif* sites, spontaneous induction, lysogenic conversion, Asemoviridae

## Abstract

Bacteriophages are the most abundant biological entities in the oceans and play key roles in bacterial activity, diversity and evolution. While extensive research has been conducted on the role of tailed viruses (Class: *Caudoviricetes*), very little is known about the distribution and functions of the non-tailed viruses (Class: *Tectiliviricetes*). The recent discovery of the lytic *Autolykiviridae* family demonstrated the potential importance of this structural lineage, emphasizing the need for further exploration of the role of this group of marine viruses. Here, we report the novel family of temperate phages under the class of *Tectiliviricetes*, which we propose to name “Asemoviridae” with phage NO16 as a main representative. These phages are widely distributed across geographical regions and isolation sources and found inside the genomes of at least 30 species of *Vibrio*, in addition to the original *V. anguillarum* isolation host. Genomic analysis identified *dif*-like sites, suggesting that NO16 prophages recombine with the bacterial genome based on the XerCD site-specific recombination mechanism. The interactions between the NO16 phage and its *V. anguillarum* host were linked to cell density and phage–host ratio. High cell density and low phage predation levels were shown to favor the temperate over the lytic lifestyle for NO16 viruses, and their spontaneous induction rate was highly variable between different *V. anguillarum* lysogenic strains. NO16 prophages coexist with the *V. anguillarum* host in a mutualistic interaction by rendering fitness properties to the host, such as increased virulence and biofilm formation through lysogenic conversion, likely contributing to their global distribution.

## 1. Introduction

Bacteriophages are the most abundant biotic agents in the ocean [1], having a major impact on bacterial mortality, diversity and global biogeochemical cycling [2]. While some phages seem to be endemic to specific environments [1], other phages are cosmopolitan [3,4] and distributed across large distances and among different biomes [5]. Therefore, phage diversity can be equally high when examined on a local scale as on a broader, regional or global scale [6]. A bacteriophage infection may either result in lysis of the host (lytic infection) and release of new virions or in the integration of the phage genomes as a prophage in the host (lysogenic infection) [7,8]. Since prophage-encoded genes may improve host fitness [9], the outcome of phage infection as lytic or lysogenic is important for the implications of phage infections on their host communities. Bacteriophages are traditionally grouped according to their morphology and genome type (e.g., RNA, DNA) [10,11], while more modern molecular tools were suggested for a more unified taxonomic classification [12]. Classification of structural lineages is the most reliable method since it better illustrates the evolutionary pathway of the viruses [13,14,15].

The lineage of tailed dsDNA viruses is mainly represented by prokaryotic viruses such as the entire class of *Caudoviricetes*. The class of *Tectiliviricetes* are clustered in the non-tailed structural lineage [13,16]. However, exceptions were reported as, for instance, in the case of flavophage FLiP, which is a member of the non-tailed lineage based on its DJR architecture even though it has ssDNA genome [17]. The non-tailed phages PRD1 with a 15 kb dsDNA linear genome [18] and PM2 with a 10 kb dsDNA circular genome [19] are representative members of *Tectiliviricetes*. For the *Tectiviridae* family (Order: Kalamavirales), 12 different species in five genera have been described [20,21]. However, for *Corticoviridae* (Order: Vinavirales), phage PM2 has been the main focus for research in protein composition, penetration process, etc. [22,23,24], and in the 50 years since its original isolation, only one additional 10 kb *Pseudoalteromonas*-specific potential *Corticoviridae* family member has been sequenced [25]. However, references of PM2-like morphology viruses can be found even for phylogenetically distant isolation hosts such as *Microbulbifer* and *Pseudanabaena* with genomes of 48.5 and 137 kb, respectively [26,27].

The recent work of Kauffman et al. [28] unraveled a new family (*Autolykiviridae*) of lytic, 10 kb non-tailed *Vibrio* predators, whose presence and role in the oceans have so far been overlooked. Coupling that with the previously reported wide presence of putatively inducible PM2-like prophages in the genomes of aquatic bacteria [29] suggested that a significant proportion of the non-tailed viral elements may still go unnoticed from the general analyses of marine phages. Certain lab-related biases were identified as the main reasons for the systematic omission from the studies of environmental phages. Sensitivity to chloroform, low buoyant density, requirement for protease treatment and long incubation time for plaque formation are features that characterize non-tailed viruses [28]. These features are typically not compatible with standard protocols for phage isolation, and likely part of the explanation for the limited data available on prokaryotic non-tailed viruses from the marine environment [30,31]. Although electron microscopy of non-tailed vibriophages and virus-like particles (VLPs) are sporadically reported in the literature, little work has been implemented on characterizing further their life traits and genome [32,33,34,35,36]. Sequencing is of utmost importance in order to confidently detect novel DJR viral elements and avert false positive results, as almost happened in the case of lactic acid bacteria (LAB) starter cultures. Large numbers of spontaneously induced tailless prophages were observed in LAB cultures; however, it was later shown that those were just phenotypic variants of the P335 siphovirus morphotype with disrupted phage tail encoding genes [37,38].

In the current study, we identify and characterize a novel family of inducible non-tailed prophages, designated as “Asemoviridae” (Asemos (Greek, άσημος): Unknown, fameless, obscured), which are prevalent in the *Vibrio* genus on a global scale. Furthermore, using the recently published, representative phage NO16 [39] and its fish pathogenic *V. anguillarum* host, we assess in vitro the variability, dynamics and mechanism of integration, as well as the spontaneous induction of the NO16 phages in the phage–host system, and evaluate the role of cell density and phage predation in the process. Furthermore, exploring the mutually beneficial coexistence with the bacterial host through lysogenic conversion and the functional role of NO16 in the community demonstrated that the prophages contribute to host cell functional properties, such as virulence and biofilm formation.

## 2. Results

### 2.1. Characterization of NO16

**Figure 1:**
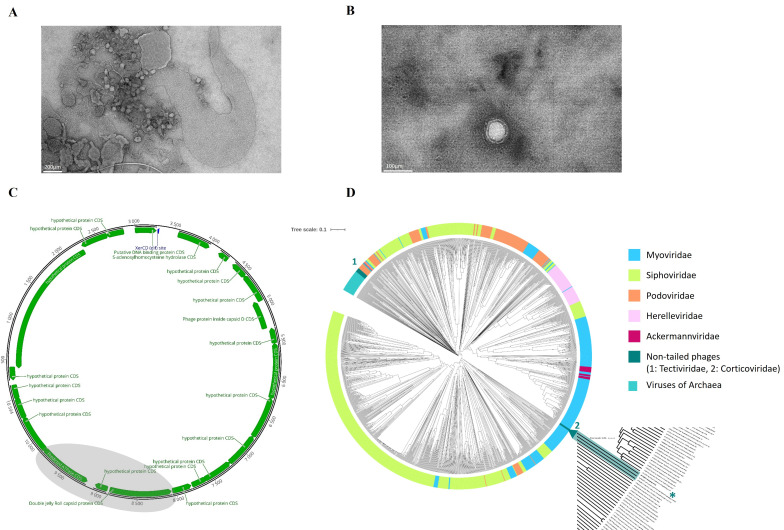
(**A**) Successful infection and consequent cell lysis of *V. anguillarum* strain A023 by phage NO16 under TEM, (**B**) Morphology of the NO16 non-tailed virions under TEM, (**C**) Genomic map of bacteriophage NO16 (GenBank accession no. MH730557) illustrating the 23-gene content of the novel phage. The viral core genes, DJR MCP and ATPase, are slightly shaded while the attributed functions of all 23 genes are listed on the side. (**D**) Dendrogram generated by GRAViTy (Database selection: DB-B: Baltimore Group Ib—Prokaryotic and archaeal dsDNA viruses). The major viral families are color coded and the phylogenetic clade, which includes the novel phage NO16, is highlighted and magnified in order to illustrate its single monophyletic taxon with only one member, marked with an asterisk *.

Screening a collection of 25 different *V. anguillarum* strains [40] showed that only strain A023 was sensitive to bacteriophage NO16, and this strain was, therefore, used as phage proliferation host. The morphology of phage NO16 under transmission electron microscopy (Figure 1A,B) revealed non-tailed virions of approximately 80 nm in diameter, which resembled the corticovirus PM2 [41] and the recently described phages of *Autolykiviridae* family [28]. The viability of the NO16 phages was significantly affected by chloroform treatment with titers of chloroform-treated NO16 phages that were three orders of magnitude lower than untreated controls (Supplementary Figure S1A). From the one-step growth curve of NO16, latency time and burst size of 30 min and 31 virions per cell, respectively, were calculated (Supplementary Figure S1B). Increasing the multiplicity of infection (MOI) also increased the initial inhibition of the *V. anguillarum* strain A023 host (Supplementary Figure S1C), whereas the total duration of inhibition (~5 h) and total viral production (~10^8^ PFU mL^−1^) was unaffected by the initial MOI (Supplementary Figure S1D).

The genome size of vibriophage NO16 is 10,594 bp (GenBank accession no. MH730557) [39], which makes it one of the shortest dsDNA bacteriophages that has been described in the literature (Figure 1C). Although the majority of the predicted phage genes are of unknown function, in silico predicted roles were attributed to five out of the 23 genes by combining RAST [42] with phyre2 [43], as previously reported [39]. DNA-binding protein (gene 6,fNO16_0006), S-adenosylhomocysteine hydrolase (gene 7, fNO16_0007), phage hydrolase (gene 11, fNO16_0011), double-jelly roll major capsid protein (DJR MCP) (gene 19, fNO16_0019) and ATPase (gene 21, fNO16_0021) were the genes with a predicted function (Supplementary Table S3), and their potential implications for the phage biology were examined.

In order to identify the taxonomy of NO16, a phylogenetic dendrogram against the Baltimore Group Ib—Prokaryotic and archaeal dsDNA viruses was constructed (Figure 1D). The phylogenetic position of NO16 is most related with, yet very significantly distinct from, the sole non-tailed corticoviruses that are included in the database. This suggests that vibriophage NO16 is a member of a novel family, which is classified as a cohesive, monophyletic taxon in a major proteome-based clustering tool such as GRAViTy, following the main principles that were recently suggested as the roadmap for genome-based phage taxonomy [44]. Based on the concatenated aminoacid sequences of DJR and ATPase encoding genes, a ML-based phylogenetic tree confirmed that the NO16 family bacteriophages form a distinct, monophyletic clade that is clearly separated from other non-tailed phage representatives: corticoviruses, autolykiviruses and tectiviruses (Supplementary Figure S2).

### 2.2. Genetic Diversity and Distribution of NO16 Viral Elements in Vibrio Genomes

An initial analysis of phage NO16 distribution in the GenBank generated certain hits (E-value: 0) in chromosome 2 of *V. anguillarum* strains 87-9-116 and NB10, isolated from Sweden and Finland, respectively [40], with identical query coverage and identity of 98% and 99.65%, respectively. This indicated that NO16 is a prophage, which is integrated in bacterial chromosomes and was not identified by regular prophage finding tools e.g., PHASTER [45]. A third hit in chromosome 2 of *V. anguillarum* VIB18 with 99.91% identity yet with a low coverage of 52% raised additional questions regarding the potential existence of phage NO16 elements in more *V. anguillarum* strains, which may have been accidentally omitted from the submitted genomes. Subsequent access to the original contigs unraveled assembly gaps in the chromosomes submitted to NCBI and identified NO16 prophages in genomic parts of 10 genomes that were not included in the submitted versions. All available contigs of *V. anguillarum* genomes [40] were re-submitted and the new accession numbers JAHGUG000000000-JAHGVD000000000 are now available in the GenBank.

The in silico results were confirmed by the spontaneous induction experiments (Supplementary Figure S3A) where the supernatants from overnight cultures of 10 bacterial strains with prophage-containing genomes could both inhibit and produce plaques on lawns of A023. Isolation, proliferation and sequencing of the induced NO16 prophages validated their genome size and content (Supplementary Figure S3B).

The induced and sequenced NO16-like phages had an identical number of genes and genomic organization as NO16 [39] but showed some nucleotide polymorphisms in particularly 2 genomic regions (NO16 genes 1–6 and 22–23), which clustered them in four closely related subgroups (Supplementary Figure S3B). However, no particular differences were detected in the generated coding regions. The sequences of the spontaneously induced NO16 phages were submitted to the GenBank under the accession numbers MZ230992-MZ231001.

**Figure 2:**
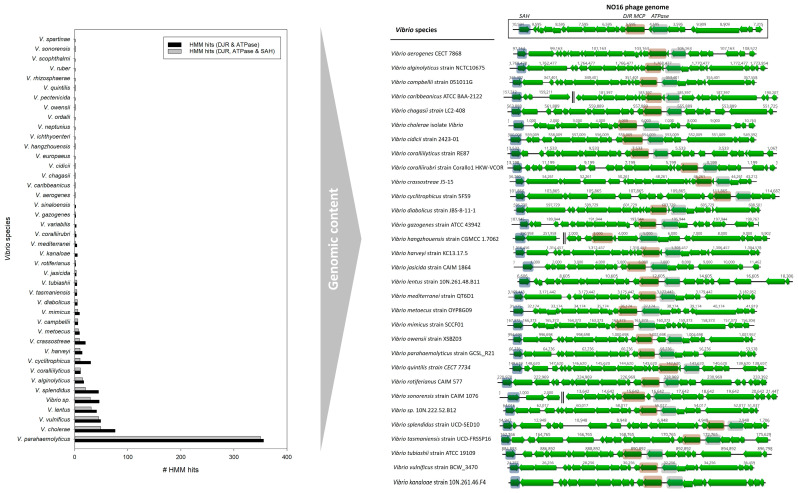
(**Left**): Successful HMM hits of DJR and ATPase and DJR, ATPase and SAH in 42 and 30 different *Vibrio* species (including the unidentified *Vibrio* sp. group), respectively, other than *V. anguillarum*. The asterisk indicates the species where NO16 phages were detected using only the DJR and ATPase core genes. (**Right**): Genomic organization of representative strains of the species where NO16 prophages were detected. The synteny of NO16-like prophage genomes are vertically illustrated, while the key genes (DJR MCP, ATPase and SAH) are consistently present and highlighted (blue: SAH, brown: DJR MCP, gray: ATPase).

The wide presence of inducible NO16-like phages in the *V. anguillarum* collection motivated the examination for this phage among the *Vibrio* genus. Searches using HMMs built on the three key phage genes ATPase, MCP DJR and SAH hydrolase generated a total of 1231, 860 and 1214 unique hits (min E-value: 0.01), respectively, in the entire *Vibrio* genome database (Supplementary Table S4). Based on the overlapping hits from all three models combined to maximize the confidence of the prophage predictions, 630 NO16-like prophage elements were found in 30 different *Vibrio* species, in addition to *V. anguillarum* (Figure 2, Supplementary Table S4). The genomic structure of the cryptic phage elements is quite syntenic across representative strains from 30 *Vibrio* species (Figure 2). The characterization of “naturally excising integrated prophages”, which was previously documented in the Vibrionaceae family [28], i.e., *Vibrio* sp. 10N.222.52.B12, *V. lentus* strain 10N.261.48.B11 and *V. kanaloae* strain 10N.261.46.F4, fit the description of the novel NO16-like viral elements while unravelling their global and intraspecific prevalence.

Our entire *Vibrio* database contained 106 different species, of which 23 accounted for 97% of the included sequences (8507 out of 8768). Overall, 19 out of the 23 most prevalent *Vibrio* species (83%) carried the NO16-like prophages, representing all available sample types: water, human, seafood and even terrestrial animal for two *V. parahaemolyticus* sequences. Moreover, the NO16-like prophages showed a global occurrence without any obvious patterns in geographical distribution or isolation source (Supplementary Figure S4).

**Figure 3:**
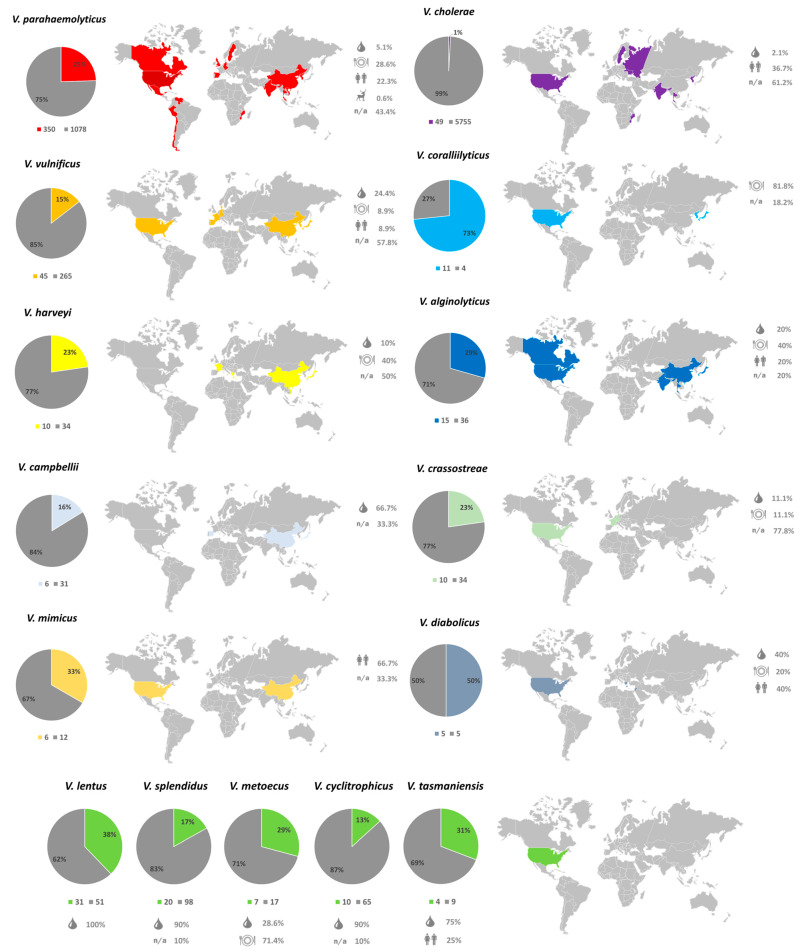
Fifteen different *Vibrio* species with more than one strain. The percentage in the colored piece of each pie chart corresponds to the NO16-containing sequences and geographic location is painted on the map. Information on isolation sources (marine, human, seafood, terrestrial animals or non-available) are also reported in the legend.

The *Vibrio* database is heavily skewed towards *V. cholerae* (5804 biosamples) and *V. parahaemolyticus* (1428 biosamples). Remarkably, fewer than 1% of the *V. cholerae* biosamples, the most abundant species in the database, were associated with NO16-like prophages (49/5804). On the contrary, the vibrios of merely marine origin harbored NO16-like phages in 13–73% of the available sequences. The vast majority of hits were recorded in the USA, in agreement with prior significant work on marine vibrios [28], representing samples from marine water, cultured seafood or humans (Figure 3). The high occurrence of NO16 in *V. parahaemolyticus* genomes unfolds its global presence and multiple isolation sources, as more than half of the total hits (350/630) were found in this species, despite it only comprising 16.5% of the *Vibrio* database. Based on this analysis, previously observed non-tailed phages such as VP2, VP7 and VP9 lytic against *V. parahaemolyticus* could presumptively belong to the NO16-like phages family, yet their sequences and characterization on the molecular level are still incomplete [32]. Representation for the rest of the marine species also demonstrated a relatively high incidence of NO16-prophage elements in their genomes. *V. coralliilyticus* is the species with the highest fraction of NO16-like prophages, with the phage elements being present in 11 out of 15 genomes (73%). Although the low number of available sequences hampers a generalized conclusion, the previous indications of 10 kb non-tailed viral elements associated with *V. coralliilyticus* obtained from gel electrophoresis studies of phage YB2 [33] or electron microscopy of phage RYC [34] imply that these phages could potentially be viruses related to the NO16 family. However, lack of genomic data renders such a conclusion rather speculative.

The inducible, cryptic NO16 bacteriophages that are widely present in *V. anguillarum* provide insights on a novel bacteriophage family with the suggested name of “Asemoviridae”. To our knowledge, this is the first sequenced and characterized temperate 10 kb non-tailed bacteriophage whose naïve host is also isolated and fully characterized. The ability to handle the original host in the lab allowed further studies on the regulation of lysis/lysogeny switch and the potential properties that the prophages may render to their hosts (lysogenic conversion).

### 2.3. Lysogenization and Development of Resistance

**Figure 4:**
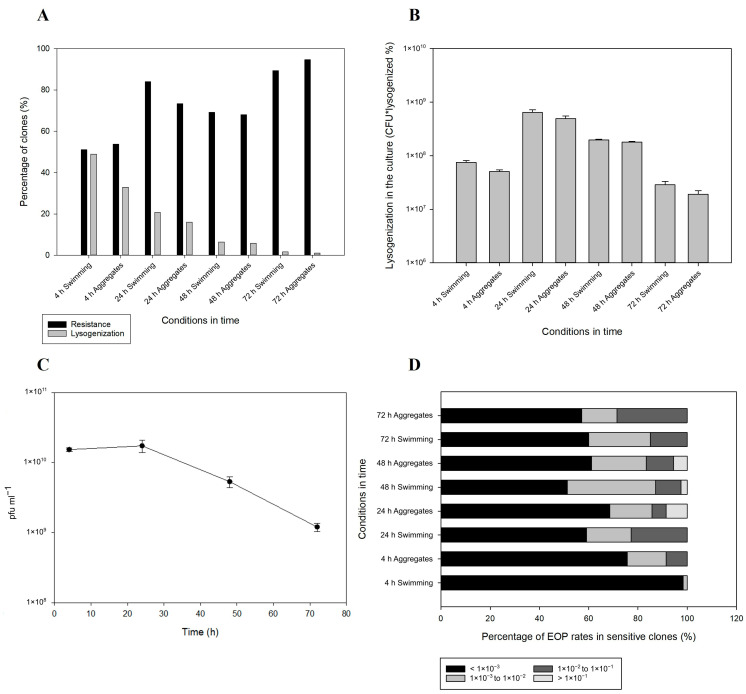
(**A**). Progress of lysogenization and resistance development over 72 h where potential responses between free-swimming and aggregate-forming subpopulations were assessed, (**B**). Number of lysogenized CFUs in the NO16-infected V. anguillarum strain A023 cultures, (**C**). Gradual decrease in free viruses over the 72 h experiment, (**D**). EOP percentages of the 306 NO16-sensitive bacterial clones over the 72 h experiment, in both free-swimming and aggregate-forming subpopulations. The values are means ± standard deviation of the three replicates.

The prevalence of cryptic 10 kb non-tailed prophage elements in vibrios indicates a looming mobility for NO16, which can be assessed by the integration potential of the viruses in *V. anguillarum*. In order to assess the lysogenization rates of the NO16 phage, as well as the development of resistance against the bacterial host over time, a large-scale bacterial isolation effort of roughly 1500 clones during a phage exposure experiment was conducted, including determination of potential differences in lysogenization between free-swimming and aggregate-forming subpopulations. In both sample types, lysogenization began very early in the infection process, reaching the highest rate at 4 h incubation (Figure 4A). Both lysogenized (40.9%) and resistant (52.4%) fractions during the early stage of infection were similar among free-swimming and aggregate fractions. Moreover, most of the lysogenized bacteria were resistant to NO16, pointing to prophage-mediated immunity against re-infection [46]. However, 45% and 38% of the lysogens after 4 and 24 h, respectively, regardless of habitat, were still susceptible to the phage. The lysogenization rate at that stage was 20% higher in the free-swimming bacteria compared to the aggregates. During the following stages of the infection, however, no differences between bacterial habitats were reported. Lysogenization continuously decreased over time, reaching 1% after 72 h, whereas resistance reached 95% by the end of the experiment (Figure 4A). The decrease in lysogenization levels over time in the NO16-infected *V. anguillarum* cultures seemed to follow the pfu counts; however, a substantial number of lysogens were maintained during the infection (Figure 4B,C). Hence, during the stationary phase, there seemed to be a selection for resistance mechanisms other than prophage-mediated immunity, as the fraction of lysogens was decreasing while the isolates maintained resistance. Interestingly, the phage-sensitive clones of the bacterial population isolated during the experiment had reduced phage susceptibility upon phage exposure, relative to the wild type. According to EOP, 75 to 98% at the early stage (4 h) and 50 to 65% after 24 h of the sensitive strains had at least a 1000-fold decrease in sensitivity to NO16 infection compared to the wild type (Figure 4D). Out of the entire experiment, 306 clones were sensitive and only 7 of them recorded a less than 10-fold decrease in sensitivity, indicating that a subpopulation of sensitive clones allows for the proliferation of new virions.

### 2.4. Prophage Integration and Spontaneous Induction Dynamics

**Figure 5:**
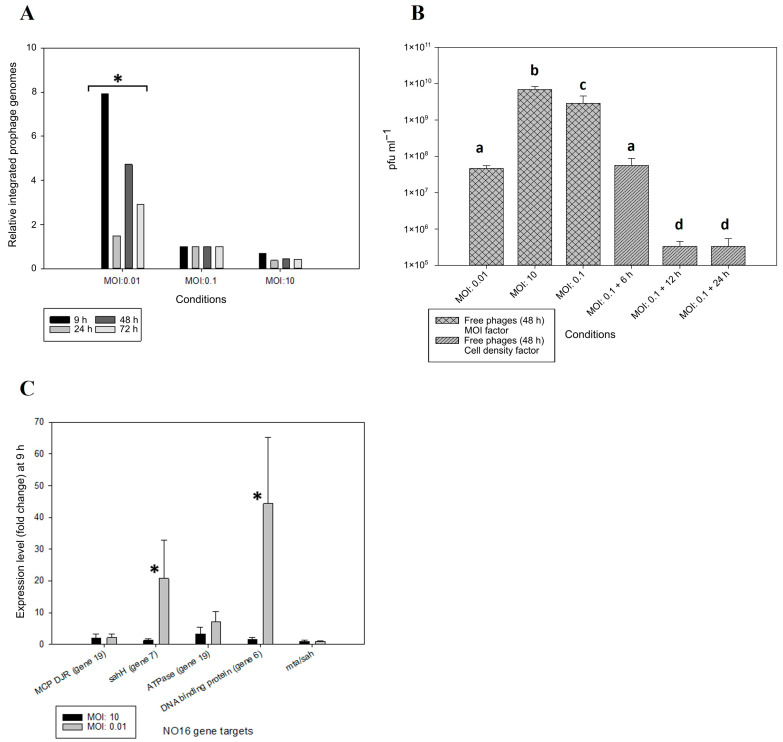
(**A**) Relative integrated NO16 prophage genomes (prophage per bacteria genomes) among different MOI (0.01, 0.1, 10) according to the quantitative assessment of the prophage–host integration sites, (**B**) Abundance of free phages 48 h post infection at different MOI (0.1, 1, 10) and cell densities (addition of 6 h, 12 h and 24 h host’s spent supernatant) conditions, (**C**) Expression levels of NO16-related genes at the early stage of infection (9 h) under different MOI (0.01, 10). The values are means ± standard deviation of the three replicates. (a, b, c, d: different groups of statistical significance, *p*-value < 0.05; * statistical significance, *p*-value < 0.05).

The integration of NO16 into the host genome and the spontaneous induction of NO16 prophages was examined at various MOIs and cell density levels, respectively, in order to assess the potential role of phage predation pressure or cell density-related factors on prophage mobility. Using a moderate MOI of 0.1 as reference point to quantify the relative integration frequency in the host, NO16 was found to integrate at significantly higher rates at a lower MOI of 0.01, with 8-, 1.5-, 5- and 3-fold higher integration at time points 9, 24, 48 and 72 h, respectively. On the contrary, higher MOI (MOI: 10) consistently led to lower integration rates compared with MOI: 0.1 (Figure 5A). The accumulation of phages 48 h post infection decreased with increasing initial MOI, and >100-fold more free phages were detected at MOI 10 than at MOI 0.01 (Figure 5B). This suggested a higher fraction of phages integrating in the host genome at a low compared to a high initial MOI, as was also evident from the integration experiment (Figure 5A). During early stages of infection (9 h), the two NO16 genes (genes 6 and 7), which encode DNA-binding domain-containing proteins and are transcribed in opposite direction to the rest of the genome, were shown to be 44- and 21-fold more expressed, respectively, in low than in high MOI conditions (Figure 5C), in agreement with the higher lysogenization rates at low MOI, supporting their tentative function as repressors of the lysis/lysogeny switch.

The effect of cell density on lysogenization in response to the addition of spent supernatant from the naïve *V. anguillarum* strain A023 culture at MOI 0.1 showed that the accumulation of infective phages decreased with the increasing age of the added supernatant (Figure 5B), suggesting that a conditioned medium from high cell density (HCD) cultures promoted the integration of the free phages. Overall, assuming that the decrease in accumulation of free phages reflects integration and lysogenization of susceptible hosts (Figure 5A,B), these results suggest that low MOI (i.e., high relative cell density) and matured conditioned medium (i.e., high concentrations of metabolites/signal molecules present at a high absolute cell density) promote the integration of NO16 phages.

**Figure 6:**
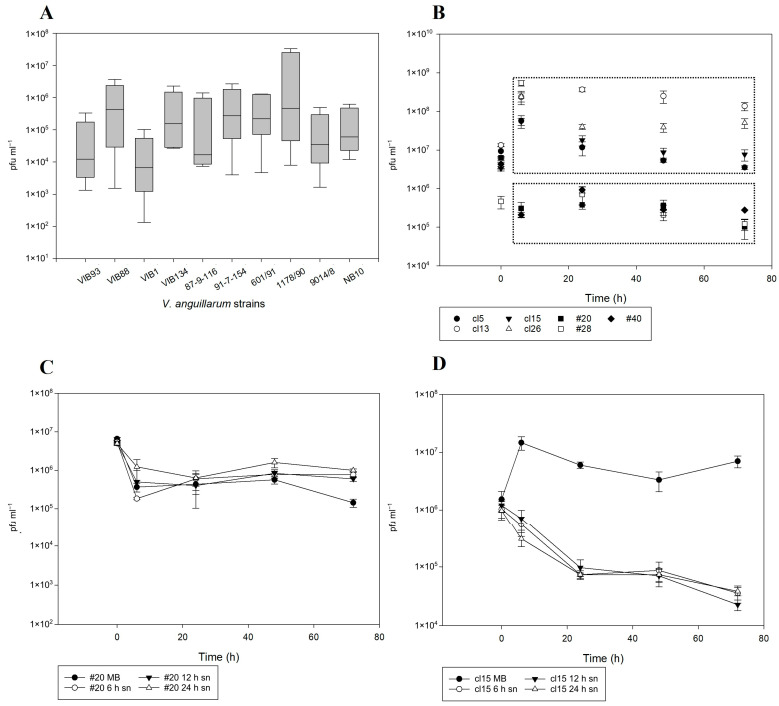
(**A**) Spontaneous induction of NO16 phages from 10 different V. anguillarum strains with considerable variations over 72 h, (**B**) Seven different lysogenized A023 clones that form two different groups based on their spontaneous induction rates over 72 h: the upper box corresponds to the high whereas the lower box to the low induction rate clones, (**C**) Spontaneous induction of lysogenized A023 clone #20 remains unaffected by different cell density levels (addition of 6 h, 12 h and 24 h host’s spent supernatant), (**D**) Spontaneous induction of lysogenized A023 clone cl15 is inhibited by increased cell density (addition of 6 h, 12 h and 24 h host’s spent supernatant). The values are means ± standard deviation of the three replicates.

The spontaneous induction dynamics of phage NO16 were examined both in the *V. anguillarum* strains in which NO16-like phages were present originally (Supplementary Figure S3A) and also in seven A023 experimentally lysogenized clones. In the former group, prophage induction varied considerably between strains with differences in observed pfu production ranging from 1 × 10^2^ to 3 × 10^7^ pfu mL^−1^ (Figure 6A). The presence of prophages in lysogenized A023 clones cl5, cl13, cl15, cl26, #20, #28 and #40 was initially detected both by PCR on the integrated prophages and by pfu formation on the *V. anguillarum* strain A023 bacterial lawn. The seven NO16 lysogenized clones were sequenced and submitted in the GenBank under the accession numbers JAHKER000000000-JAHKEX000000000. The dynamics of the spontaneous induction among different lysogens were also variable, supporting the observed strain-specific variability of prophage induction on the environmental strains. Based on the induction pattern, the seven lysogenized clones clustered in two groups: Group 1 including strains cl5, cl13, cl15, #28 and cl26 with a high induction rate (4 × 10^5^–3.5 × 10^8^ phages produced per mL) and group 2 with strains #20, #28 and #40 with an initial decrease in phage abundance, and only 4.5 × 10^3^–4 × 10^5^ phages produced mL^−1^ (Figure 6B). The lysogenized strains cl15 and #20 were selected as representatives of the high and low induction group, respectively, for further examination of their response under various cell density conditions, emphasizing their different spontaneous induction behavior. In the case of low-induction strain #20, the addition of the conditioned medium did not affect prophage induction, and a decrease in induced phages similar to the initial induction experiment was observed in all treatments (Figure 6C). For the high-induction strain cl15, however, the presence of the conditioned medium inhibited the release of new virions by at least 2 log units (Figure 6D), leading to a response in prophage induction similar to the Group 2. Overall, these strain-specific differences in prophage induction suggested that the regulation vary between strains and may involve quorum sensing or other cell-density related factors.

### 2.5. Genomic Mechanism of Integration and Induction

According to comparative genomic analysis among the NO16-like sequenced prophages and the resubmitted contigs of lysogenized *V. anguillarum* strains, it seems that the mechanism of phage integration in the bacterial host genome may be driven by a XerCD (chromosomally encoded tyrosine recombinases) site-specific recombination mechanism. In *Escherichia coli*, XerCD recombinases recognize a 28-bp motif, called *dif* site, and through site-specific recombination, they untangle circularized chromosomal dimers [47]. By carrying *dif*-like sites, filamentous phages such as CTXφ utilize host-encoded XerCD to recombine with its host genome, and similar *dif*-like sites were also found in the *V. anguillarum* filamentous phage VAIφ [48,49,50] (Supplementary Figure S5). The XerCD-*dif* site-specific recombination process is facilitated by DNA translocases through ATP hydrolysis, and homologs of those enzymes are typically found in the vast majority of sequenced bacteria [47].

### 2.6. Role of NO16 Prophages for Bacterial Functional Properties

**Figure 7:**
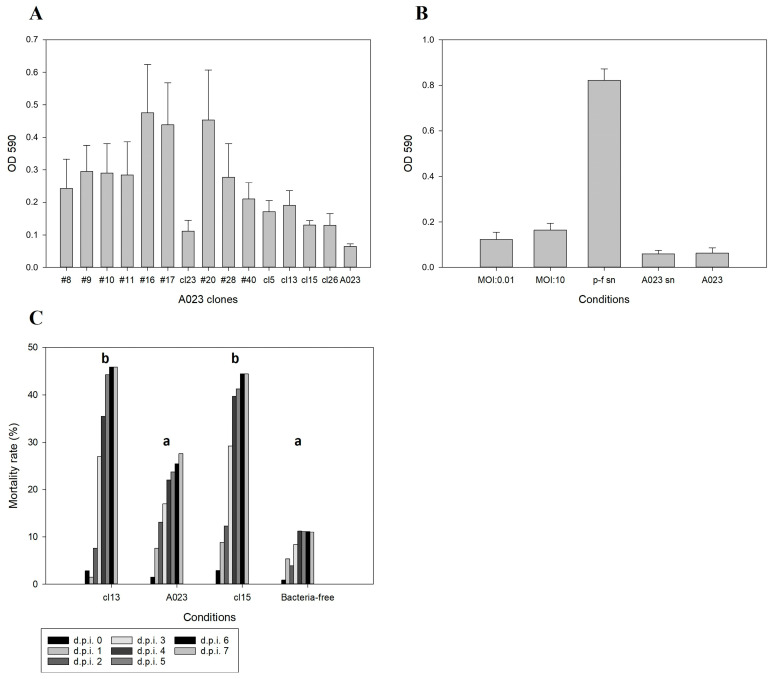
(**A**) Biofilm formation of lysogenized (#20, #28, #40, cl5, cl13, cl15, cl26) and non-lysogenized (#8, #9, #10, #11, #16, #17, cl23), but exposed to NO16, A023 clones after 48 h. A023 was used as control. (**B**) Biofilm formation of A023 under low (MOI: 0.01) and high (MOI: 10) phage predation, as well as in the presence of host’s spent supernatant (A023 sn) and supernatant from bacteria–phage lysis event (p-f sn). A023 was used as control. (**C**) In vivo mortality rates of *S. aurata* larvae infected by A023 lysogens (cl13 and cl15), wild type (A023) and non-infected (bacteria-free) condition as negative control, over a 7-day period. Letters a and b indicate statistically significant groups (Dunn’s method versus control). The values are means ± standard deviation of the three replicates.

The wide prevalence and mobility of NO16 phages raises questions about their potential role in aquatic bacterial communities, and their potential contribution to biofilm formation and virulence was assessed. Biofilm formation was quantified for the seven lysogens (cl5, cl13, cl15, cl26, #20, #28 and #40) but also for seven more strains, which were exposed to phage predation but were not lysogenized (#8, #9, #10, #11, #16, #17 and cl23). The control wild type strain A023 was a poor biofilm former, and there was a statistically significant increase in biofilm formation for all the lysogens with the exceptions of cl15 and cl26 at 48; however, all strains were significantly better biofilm formers in at least one sampling point (Figure 7A). The addition of the phage NO16 significantly increased the biofilm formation for A023 strain at 48 and 72 h at both low and high MOI. In addition, while the supernatant that was harvested from naïve A023 culture (A023 sn) had little effect on biofilm formation, the phage-free supernatant that was harvested from the A023/NO16-infected culture (p-f sn) significantly increased biofilm formation (Figure 7B). No direct link between prophage induction rates and biofilm formation was observed. Overall, the presence or previous exposure of NO16 in the bacterial population seemed to promote biofilm formations regardless of it being integrated in the chromosomal genome or not. In addition to biofilm formation, we assessed if lysogenized strains had obtained potential virulent properties from the prophages by challenging Gilthead seabream (*Sparus aurata)* larvae with the lysogenized strains cl13 and cl15 and the naïve A023 host. Over a two-week experiment, both lysogens were significantly more virulent compared to their naïve counterpart and the bacteria-free control treatment (Figure 7C). Strain A023 was previously characterized as low virulence among several tested *V. anguillarum* strains [40], and together with the enhanced biofilm potential, our current results suggest that lysogenic conversion by NO16 does indeed affect host functional properties.

All exposed but not lysogenized clones were also sequenced and submitted in the GenBank under the accession numbers JAHKEJ000000000-JAHKEQ000000000.

## 3. Discussion

The interactions between bacteriophages and their hosts play key roles in bacterial ecology, diversity and evolution in the marine environment; however, we still know very little about their diversity and distribution. The current discovery and characterization of a new family of non-tailed marine phages with a global distribution as a prophage among 30 *Vibrio* species emphasizes the vast importance of these particular phages in shaping the genetic and functional properties of their bacterial hosts and illustrates their dissemination potential across bacterial species and large spatial scales. The NO16 phages (“Asemoviridae”), which were originally isolated from *V. anguillarum* and, subsequently, shown to be present as a prophage in the entire Vibrionaceae family, have so far been undetected by traditional bioinformatic prophage detection tools [45,51,52]. The recent discovery of a new family of lytic non-tailed DJR capsid vibriophages, *Autolykiviridae*, has demonstrated that DJR viruses are far more diverse than previously recognized, representing important killers of marine Vibrios [28]. The “Asemoviridae” show very little genetic similarity to the *Autolykiviridae,* even among their signature core genes, such as gene 19 (MCP DJR) and gene 21 (packaging ATPase). Kauffman et al. [28] have already addressed the presence of naturally excising prophages in Vibrionaceae, which were not classified in the *Autolyikiviridae* family, corroborating the existence of more than just three recognized non-tailed families. BLAST failed to generate any substantial genomic overlap between any genes of the two viral families, and we, therefore, proposed that the “Asemoviridae” represents a novel family of phages. Due to the underrepresentation of non-tailed phages in the database, there is a special reference to them in the recently published roadmap for genome-based phage taxonomy, according to which we need new criteria to be tailor made for such special groups [44]. However, the fundamental principle that rules the phage taxonomy roadmap is confirmed in this case for “Asemoviridae”, since the generated GRAViTy classifies them as a distinct, cohesive and monophyletic taxonomic unit. The statistical robustness of the phylogenetic tree that was constructed, including representative viruses from the class of *Tectiliviricetes*, corroborates the distinct taxonomic position of this novel phage family. The low genomic and amino acid similarity among the DJR protein among *Autolykiviridae*, *Corticoviridae, Tectiviridae* and “Asemoviridae” [39] dictates that the distinction of families is directly reflected on the structural level and further expanded to the rest of the genomic content. Although demarcation criteria for non-tailed viruses are not yet as standardized as they are for their tailed counterparts, the articulate and recent roadmap for genome-based phage taxonomy infers that common principles are to be shared among bacterial viruses with the additional consideration for case-specific amendments [44]. Furthermore, their temperate lifestyle and wide dispersal among Vibrios originating from environmental, human, seafood, terrestrial animal sources suggest that this family of phages is not only an important killer of marine Vibrios but also contributes to host performance when integrated into their genome. This suggestion was strongly supported by our experimental verification that the “Asemoviridae” phages are dynamic components of 40% of the fish pathogenic *V. anguillarum* strains in our collection, with positive implications for key functional properties, such as virulence and biofilm formation by the lysogenized strains.

Beyond the *Vibrio* genus, there are only a few known examples of non-tailed phage isolates, such as the lytic phages PM2 [41], Cr39582 [25] and FLiP [17], which infect *Pseudoalteromonas* and *Flavobacterium*, respectively, with poor to no genomic similarities to the new family of “Asemoviridae”. The identification of viral “self” genes was useful for exploring genetic relatedness among DJR phages [29]. The analysis of these genes have supported the presence of major capsid protein and packaging ATPase as essential genes for all corticoviral elements, such as PM2 and revealed hidden PM2-like elements inside the genomes of aquatic bacteria [29]. Although these elements were previously reported as presumably circular, it was not determined if they are inducible, as demonstrated for “Asemoviridae” in the present study. Previous observations of inducible DJR elements from *V. kanaloae* and *V. cyclotrophicus* by Kauffman et al. [28] suggested that these phages were indeed mobile. In the current study, however, the availability of a naïve, susceptible *V. anguillarum* strain A023 host was key to validate circularity and examine induction and integration potential of the “Asemoviridae” phages. Coupling current and previous findings suggests that also other non-tailed DJR phages such as PM2, which has long been characterized as a lytic phage [19,41], may have a so far undetected lysogenic lifestyle under the right infection conditions of a susceptible *Pseudoaltermonas* host. The in silico synteny of the PM2 genome with chromosomal parts of *P. piscicida* and *P. lipolytica* are indications supporting an undiscovered temperate PM2 lifestyle, as was shown for the temperate PM2-like phage Cr39582 [25]. A presumptive 28-bp *dif* site was found in the genome of PM2 (CGTGCTTACGATT-TATA-TTATGTTAAAT), though with 8 mismatches compared to the NO16 *dif* site.

The wide prevalence of NO16 phages was validated by the construction of HMMs, which were based on the viral “self” genes of the non-tailed elements. Probabilistic models for protein homology and structure prediction were previously used for both the tailless dsDNA corticoviruses and tectiliviruses [29,53,54], while core genes such as DJR MCP were recently used as bait to successfully unravel their diversity and prevalence in bacterial genomes and metagenomes [54,55]. In the current study, we used the overlapping results of three individual HMMs to identify the presence of NO16 viruses in *Vibrio* genomes to ensure high confidence of the prediction, resulting in 630 genomes containing all three genes. If single individual models were used, the estimated prevalence exceeded 1200 genomes, and we, therefore, believe that the 630 prophage hits represent a conservative estimate. From an evolutionary perspective, such probabilistic models have significant value because although the majority of viral “self” genes encode structurally conserved proteins, detection of homology in both their amino acid and genomic level is rather challenging.

Our results add to the emerging consensus of a high prevalence of lysogeny in marine bacteria [56,57]. A recent screening of 1778 marine bacterial genomes demonstrated that on average 17.7% were lysogenized with at least one prophage across all phyla, and within the *Vibrio* genus, 29% of the 91 published genomes were lysogenized [57]. In our study, 180 of the 679 analyzed *Vibrio* genomes of confirmed marine origin (26.5%) contained the NO16 prophage, suggesting that the frequency of marine vibrios, which contain the “Asemoviridae” prophage is similar to the total frequency of lysogenized vibrios detected using traditional prophage-finder tools detected.

Wide geographical coexistence patterns of bacteria and their corresponding bacteriophages were previously described, not only for *Vibrio* phages [3,58] but also for phages infecting other genera [4]. The global presence of asemoviruses as prophages strongly supports previous suggestions that DJR bacteriophages are highly overlooked [13,28] and emphasizes the potential for the discovery of new viral families within the DJR viral lineage.

In the *V. anguillarum* strain A023 and phage NO16 system, lysogenization during the early stages of infection affected almost half of the population, with a 20% higher lysogenization rate in the free swimming bacteria compared to the aggregates. This suggested that aggregate-associated bacteria were partially protected from infection, as previously reported for *V. anguillarum* [59]. Over time, there was a strong selection for phage-resistant bacteria at the expense of the lysogens, and phage production was likely maintained by infection of a small fraction of susceptible bacteria and by induction of prophages. The results demonstrate that phage exposure drives a diversification of the host population and leads to co-existence of phage-sensitive non-lysogens, phage-resistant and sensitive lysogens and phage-resistant non-lysogens, which likely contributes to the successful dissemination of the phage.

Interestingly, phage integration was negatively correlated to the multiplicity of infection, as both frequency of lysogenization and expression of selected prophage genes in the host were significantly higher at low than at high MOI. Accordingly, the reduced accumulation of free phages at low MOI in the infection experiment indicated a higher integration rate at low MOI than at high MOI. The reduced accumulation of phages at low MOI could also reflect an increased rate of superinfection or decay of the produced phages in these cultures. However, apart from the initial differences in phage abundance the experimental settings were identical. Together these results point to phage integration and prophage induction being favored at low and high MOI, respectively. However, an alternative hypothesis that the MOI dependent lysogeny rate could be a matter of the host being able to “capture” a low number of phages via XerCD, whereas a high number of phages may overwhelm the system, cannot be ruled out. Previous knowledge on the lysis–lysogeny regulation mainly stems from experiments with temperate phages infecting *E. coli*, such as λ, Mu, P1 or N1 [60]. These have generally reported the opposite trend, i.e., high MOI favors lysogeny in these phages, which was interpreted as a strategy to reduce the risk of unsuccessful lytic infection of the next phage generation during conditions, where phages outnumber their bacterial hosts (i.e., high MOI) [61,62,63]. In the marine environments, however, lysogeny is considered to be less prevalent in oligotrophic waters with high virus–bacteria ratios (VBR) [56]. The large range in VBR (from 3 to 160) observed across marine environments [64] and decline in VBR at increasing microbial densities have led to the so-called Piggyback-the-Winner model [65], suggesting an increased prevalence of lysogeny at high cell densities as the key driver of this relationship between VBR and cell density. The current study also indicated a correlation between cell density and the induction of NO16 prophages, since the accumulation of free phages from induction in *V. anguillarum* was negatively correlated with age of the culture supernatant added to the lysogens. Addition of cell-free supernatant from high cell-density cultures (24 h and 6 h cultures of then non-lysogen) led to significantly lower accumulation of free phage particles than cultures without supernatant or supernatant from low cell density (LCD) cultures, suggesting that extracellular metabolites, such as QS signal molecules, in the spent medium could reduce prophage induction [66]. Previous studies have reported that host cells through QS may regulate the lysis–lysogeny switch and trigger prophage induction [67,68]. Although it is not known if QS regulates NO16 prophage induction, our results support that NO16-like prophages favor lysogeny at high cell density conditions in *V. anguillarum*, presumably to promote host fitness. [66]. This hypothesis was further supported in the current study by the observed significant increase in biofilm formation and virulence against fish larvae in the lysogenic strain compared with the non-lysogen. Together, these data support that the interaction between host and prophage is regulated both by the host density through QS pathways and by the phage–host ratio (MOI), resulting in relatively high induction and low integration frequency of prophages at LCD and high phage–host ratio, and vice versa (i.e., low induction and high integration) at HCD and low phage–host ratio. Interestingly, the response to cell-free supernatant from HCD cultures of the non-lysogenic *V. anguillarum* wild type A023 seemed to vary between prophage-bearing clones, as indicated by the differences in prophage induction in the lysogenic strains cl15 and #20 after exposure to spent medium. While this may reflect clone-specific differences in QS response, other cell-density factors, which are not yet known, may play significant roles as well. These differences in how the regulation of the lysis–lysogeny switch fits into different phage and host strategies, cell density and environmental conditions highlights the need for a deeper understanding of this process and its ecological and disease implications.

## 4. Materials and Methods

Abridged Methods are provided below; details and additional information are provided in the extended Methods in the SI. All experiments were conducted in triplicate.

### 4.1. Bacterial Strains and Growth Conditions

The strains used in this study were collected from clinical incidents of vibriosis outbreaks at different fish farms in numerous countries around the world. All bacterial strains were previously sequenced and classified to the species of *V. anguillarum*, and their virulence and pathogenicity traits were characterized [40,69]. Marine broth (0.5% tryptone, 0.1% yeast extract and 2% sea salts) was used for the liquid bacterial cultures, with the addition of 1.5% agar for growth on solid medium.

### 4.2. Isolation, Purification and Proliferation of Bacteriophages

Samples for phage isolation were collected at a coastal area of the Central East mainland of Greece, and the presence of bacteriophages was assessed by the classic enrichment method [70] using a combination of four *V. anguillarum* strains (A023, 261/91, 178/90 and 601/91) [40]. The water was supplemented with 10× marine broth adjusting the nutrient concentrations according to the final volume, and the enrichment culture was incubated overnight at 25 °C with constant agitation. Samples of 1 mL were then centrifuged at 6000× *g* for 10 min, filtered through 0.22 μm pore-size syringe filters and the supernatant was spotted on the bacterial lawn of each candidate host using the double agar layer method [71]. Samples producing clearing zones on the bacterial lawn were serially diluted in SM buffer (sodium magnesium; 100 mM NaCl, 8 mM MgSO4·7H_2_O, 50 mM Tris-Cl, 0.01% gelatin, pH: 7.5), and 10 μL of each dilution were mixed with 90 μL of mid-log-phase bacterial culture and left for 5 min to allow bacteria and phage binding. Then, 1 mL melted soft agar (0.4% agar and 1% Sigma-Aldrich sea salts) was added, and it was poured into 6-well plates with marine agar. Following overnight incubation at room temperature, the wells were examined for the presence of plaque forming units (pfu). Individual pfu were picked and purified by repeating the process five times in order to create clonal phage stocks.

### 4.3. Characterization of Bacteriophages

The morphology of the phage NO16 was observed under a JEM-1010 transmission electron microscope (Jeol, Tokyo, Japan) by mounting them on negatively stained copper grids as previously described [48]. Phage concentrates were prepared by the addition of poly-ethylene glycol 8000 (PEG-8000) and sodium chloride (final concentration 10% *w*/*v* and 1 M, respectively), and the phage solutions were incubated overnight at 4 °C. Finally, the suspensions were centrifuged (10,000× *g*, 30 min, 4 °C) and the phage pellet was resuspended in 200 mL of SM buffer [72].

Latency time and burst size of the novel virions were calculated from their one-step growth curve. Phage NO16 was added to 1 mL early exponential phase *V. anguillarum* A023 culture in marine broth using low multiplicity of infection (MOI: 0.001) and incubated with agitation for 15 min at room temperature. After centrifugation at 6000× *g* for 10 min, the bacteria–phage pellet was resuspended in 20 mL fresh marine broth, samples were taken every 10 min for a total of 100 min, and infective phages were quantified as above.

To evaluate chloroform sensitivity of phage NO16, chloroform was added to the phage stock in a ratio of 1:5 followed by quantification of infective phages. Mixtures of phage and chloroform were vortexed for 6 s and incubated at room temperature for 2 h. Following centrifugation at 5000× *g* for 5 min, phage viability was tested using serial dilutions mixed with the bacterial hosts and poured into marine-agar-containing 6-well plates and compared with control incubations without chloroform [28].

The host range of the NO16 phage was assessed by spotting 10-μL drops of the phage stock on the bacterial lawns of 23 clinical *V. anguillarum* strains that were previously characterized [40] using the double agar layer method [71] (Supplementary Table S1).

### 4.4. Genomic Analysis and HMM Construction

DNA extraction of both bacteria and phage genomes was performed with Wizard Genomic DNA purification kit (Promega, Madison, WI, USA) following the manufacturer’s protocol. Purified DNA samples were sequenced by Beijing Genomics Institute (BGI) using Illumina HiSeq platform (BGI, Shenzhen, China) with paired-end read sizes of 100 bp. Library construction (TruSeq), sequencing and data pipelining were performed in accordance with the manufacturer’s protocol.

The *V. anguillarum* strains that were sequenced by Rønneseth et. al. [40] were assembled using *V. anguillarum* strain 775 as reference [69]. Although the assembly was successful, the genomic variability of vibrios led to the generation of several assembly gaps and, hence, omission of sequenced genomic information. Therefore, all previously sequenced data were re-submitted to the GenBank, as contigs, in order to include all the missing data. The bacterial genomes were annotated by NCBI Prokaryotic Genome Automatic Annotation Pipeline (PGAAP) [73].

The genome of NO16 bacteriophages was recently sequenced [39] and de novo assembled using the Geneious assembler at high sensitivity, which is included in Geneious Prime 2020.2 software bioinformatics platform. Genomic comparisons among NO16-like phages were conducted by using the NO16 phage’s genome as reference (GeneBank accession no. MH730557). BLAST https://blast.ncbi.nlm.nih.gov/Blast.cgi (accessed on 13 March 2020) was used as the first tool in order to screen for similar genomes in the non-redundant nucleotide database. Geneious Prime 2020.2 software bioinformatics platform was also used to visualize and in silico search the sequenced genomes, construct the genomic map of NO16 and explore the phylogenetic relationships with the spontaneously induced NO16-like phages from the different strains of *V. anguillarum*. Furthermore, a ML-based phylogenetic tree (RAxML; gamma blosum62; 1000 bootstrap replicates) using the concatenated aminoacid sequences of major capsid protein and ATPase encoding genes was constructed in order to determine the specific relationships among autolykiviruses, corticoviruses, tectiviruses and the group of NO16-like bacteriophages.

The detection of NO16-like prophages in the publicly available *Vibrio* genome database was conducted by constructing three individual Hidden Markov Models (HMM) [74] for the three key NO16 phage genes DJR MCP (gene 19), ATPase (gene 21) and SAH (gene 7). DJR MCP and ATPase are the viral “self” core genes [29], which are the signature genes of all the hitherto described 10 kb non-tailed bacteriophages. The third gene is a BLAST-predicted S-adenosylhomocysteine (SAH) hydrolase (gene 7), which is one out of only two NO16-genes transcribed in the opposite direction to the rest of the prophage genes and, according to its predicted protein structure, contains a DNA-binding domain. Analytical details for both genomic analysis and HMM construction are included in the Methods supplementary section.

### 4.5. Resistance Development and Lysogenization Assay

The rates of resistance and lysogenization, as well as potential factors that may affect them, were explored during NO16 phage and *V. anguillarum* strain A023 infection process, following an extensive clone picking experiment. *V. anguillarum* strain A023 was infected at the early exponential phase by NO16 phage (MOI: 0.1) in triplicate, and four samplings at 4, 24, 48 and 72 h post infection were performed. To separate the aggregated cells from the free-living cells, mild centrifugation (500× *g* for 2 min) was applied to determine the role of the bacterial habitat (aggregated or free-living) on the fraction of resistant and lysogenic cells. At each sampling point, bacteria from both swimming and aggregate samples were spread on marine agar plates for isolation of the individual colonies. A total of ~1500 isolates were transferred to 96-well plates with marine broth for subsequent screening for lysogenization and resistance against phage NO16. Prophage integration was assessed by examining the supernatant of overnight cultures of each isolate for lytic activity against a fresh bacterial lawn of *V. anguillarum* strain A023, using the double agar layer method [71]. Resistance of each individual isolate was likewise tested with spot assay using NO16 phage. All phage-sensitive isolates were recultured, and the efficiency of plating (EOP) was determined [71] as a measure of the various sensitivity levels that NO16 may cause in the bacterial population. Furthermore, samples were collected for optical density, pfu counts, cfu counts and light electron microscopy at all time points.

### 4.6. Bacteriophage Dynamics: Prophage Integration and Spontaneous Induction

The temperate life cycle of NO16 phage [39] was characterized by assessing dynamics of integration and induction of the phage, respectively. Phage integration was assessed at various MOI and cell density conditions to examine the role of phage and cell density on the lysis/lysogeny switch. *V. anguillarum* strain A023 was infected at early exponential phase (OD_600_ ~ 0.2), 3 h post inoculation from an overnight culture, at 3 different MOIs: low (0.01), moderate (0.1) and high (10), prepared in triplicate in 50-mL centrifuge tubes.

To examine the role of signal molecules or other extracellular metabolites produced during bacterial growth, additional experiments were established with conditioned media with cell-free supernatant from bacterial host cultures grown at different densities. The conditioned medium was prepared by growing 0.2 L liquid cultures of the *V. anguillarum* strain A023 for 6, 12 and 24 h, respectively, followed by centrifugation (6000× *g*; 10 min) and 0.22 μm filtration to obtain supernatant from cultures that had reached early exponential, late exponential and stationary phase growth, respectively. A mixture of conditioned media and regular marine broth at 1:1 ratio in triplicate 50-mL centrifuge tubes was used to generate three different cell density conditions, and phages were added at an initial MOI of 0.1. An additional incubation where *V. anguillarum* strain A023 was growing in regular marine broth without phages served as control. Samples were collected after 9, 24, 48 and 72 h, respectively (6, 21, 45, 69 h post phage infection) for quantification of lysogenic cells and phage production.

Identification of the phage integration site was evaluated at the molecular level after extracting the bacterial genome using the boiling method [75]. Specific primers were designed for measuring the relative integration site DNA copies and calibrating them to the DNA copies of a bacterial reference gene, *recA* (Supplementary Table S2, and Supplementary Methods). Liquid culture *V. anguillarum* strain A023 infected with the temperate phage NO16, led to the formation of lysogenized clones. For further examination of clonal diversity among lysogens, seven clones were randomly selected from 200 lysogenized colonies isolated after 24 h and 48 h incubations. Detection of lysogenized bacteria was performed from observations of spontaneous induction measured by PFU and by phage-targeted PCR using specific primers, which targeted signature phage genes (e.g., DJR MCP), and the integration sites where prophages were integrated (Supplementary Table S2). Negative controls of uninfected *V. anguillarum* strain A023 were always included in the PCR.

Spontaneous induction dynamics were evaluated for the seven confirmed lysogens. The strains were cultured on marine agar, and fresh colonies were picked and grown overnight in marine broth and, subsequently, inoculated in marine broth. Samples for optical density, pfu and cfu counts were obtained at 0, 6, 24, 48 and 72 h post inoculation to examine potential clone-specific variabilities in prophage induction. Based on the induction profiles, two representative strains were chosen for further analysis, where cell density and MOI were examined to evaluate their role in prophage induction. Regarding cell density, conditioned media from three different cell densities were applied for each strain as described above, and samples were collected for optical density, cfu counts, pfu counts and gene expression analysis after 9, 24, 48 and 72 h.

### 4.7. Quantification of Phage and Bacteria Dynamics

At each sampling point in the experiments above, several samples were taken and processed from all replicates in different conditions to assess optical density, bacterial cell counts, infective phage counts, prophage integration, and selected gene expression levels. Details of the applied methods are described in the Methods supplementary section.

### 4.8. Biofilm Formation in Lysogens and Non-Lysogens

The potential for biofilm formation was tested in 15 different strains: the wild type *V. anguillarum* strain A023, and seven lysogenized and seven non-lysogenized clones obtained after NO16 infection. Additionally, biofilm formation was also examined when *V. anguillarum* strain A023 was exposed to four different conditions: low and high MOI (0.01 and 10), bacteria-free supernatant and phage-free supernatant of lysed culture, to assess the role of potential signal molecules or other extracellular metabolites that may be produced during bacteria and phage proliferation [76]. Bacteria-free supernatant was retrieved after the 0.22 μm filtration of an overnight *V. anguillarum* strain A023. Phage-free supernatant was harvested after filtering overnight the *V. anguillarum* strain A023 and phage NO16 infected culture, first through 0.22 μm and then through Amicon (3 kDa, Milipore, Burlington, VT, USA). The biofilm formation protocol was based on the previously reported crystal violet method [59] modified to 96-well plates with 8 replicate wells for each condition. The final absorbance was measured at 595 nm using a TECAN Infinite Pro 200 microplate reader.

### 4.9. In Vivo Challenges of Fish Larvae to Assess Lysogenic Conversion

In vivo trials were performed using a previously described fish larvae method, ideal for bacterial virulence assays [40]. Briefly, good quality eggs of gilthead seabream, *Sparus aurata*, were collected and placed individually in 24-well plates (Cellstar, Greiner Bio-One, Kremsmünster, Austria). A total of 336 eggs in 14 plates were prepared and divided into three treatments (3 plates × 24 healthy eggs) and one control (5 plates × 24 healthy eggs). In the three treatments, the eggs were challenged with 10^6^ cells per mL (high bacterial dose) fresh overnight culture of the wild type *V. anguillarum* A023 and two lysogenized clones, respectively, whereas sterile SM buffer was added to the control plates. The plates were incubated at 18 °C and egg and larval mortality were monitored for seven consecutive days post infection (d.p.i.).

### 4.10. Statistics

Statistical analysis was performed to assess significant differences among different conditions and treatments that were tested in gene expression analysis, biofilm formation, in vitro and in vivo challenges. One-way and two-way ANOVA were performed following assessment of normality of the data distribution (significance: *p*-value < 0.05). All statistical analyses were performed with SigmaPlot, version 14.0, Systat Software, Inc., San Jose, CA, USA.

## 5. Conclusions

The prevalence of vibriophages that coexist inside virulent *Vibrio* genomes was extensively documented in several important human and animal pathogens [3,48,58,69], highlighting the importance of temperate phages for the functional and genetic diversity of this bacterial genus. The increased biofilm formation and virulence of NO16 prophage lysogens are direct evidence for lysogenic conversion [77], being properties that are affected by phage exposure [78]. The discovery of a new family of temperate phages, the “Asemoviridae”, with a global high prevalence and ubiquitous distribution across the *Vibrio* genus, having significant implications for host functional properties, have further emphasized the importance of DJR phages as mobile elements in marine microbial communities. The observed indications of the cell density dependent regulation of phage–host interactions, lysogenic conversion through biofilm formation and virulence, as well as the intra- and interspecific variabilities, represent key topics for further investigation to resolve the ecological and evolutionary role of the asemoviruses across marine bacterial genera. Updating viral genomic and metagenomic databases, isolation and cultivation of more “Asemoviridae” phage–host systems, and more targeted approaches on host and prophage transcriptome under specific infection conditions are future work that could provide crucial insights and further elucidate the role of these widespread “cryptic” viral elements.

## Data Availability

All generated and analyzed data are presented in the manuscript, its supplementary materials and are publicly available in open access databases.

## Supplementary Materials

The following supporting information can be downloaded at: https://www.mdpi.com/article/10.3390/ijms24043937/s1. References [43,58,79,80,81,82,83,84,85,86,87,88,89,90] are cited in the supplementary materials.
