# Supplementary material for: Staying below the Radar: Unraveling a New Family of Ubiquitous “Cryptic” Non-Tailed Temperate Vibriophages and Implications for Their Bacterial Hosts
Source: Int J Mol Sci. 2023 Feb 15;24(4):3937. doi: 10.3390/ijms24043937 (PMC9966536; doi:10.3390/ijms24043937)
Supplement: Supplementary file 1 [file ijms-24-03937-s001.zip › ijms-2111652-supplementary.pdf]

Supplementary Information for:

Staying below the Radar: Unraveling a New Family of Ubiquitous "Cryptic" Non-Tailed Temperate Vibriophages and Implications for Their Bacterial Hosts

Panos G. Kalatzis [1], Jesper Juel Mauritzen [1], Caroline Sophie Winther-Have [1], Slawomir Michniewski [2], Andrew Millard [2], Maria Ioanna Tsertou [3], Pantelis Katharios [3] and Mathias Middelboe [1,4,*]

[1] Marine Biological Section, Department of Biology, University of Copenhagen, 3000 Elsinore, Denmark

[2] Department of Genetics and Genome Biology, University of Leicester, University Road, Leicester LE1 7RH, UK

[3] Institute of Marine Biology, Biotechnology and Aquaculture, Hellenic Centre for Marine Research, Former American Base of Gournes, 71500 Heraklion, Greece

[4] Department of Biology, University of Southern Denmark, 5230 Odense, Denmark

The supplementary information includes three appendices. The first appendix contains information about additional methods and protocols. The second appendix provides details on additional figures. The third appendix describes supporting results in tables.

I. **Supplementary Methods**

II. **Supplementary Figures**

III. **Supplementary Tables (includes also 2 additional excel files)**

I. Supplementary methods

Text S1: Genomic comparisons. Genomic comparisons among NO16-like phages were conducted by using the NO16 phage's genome as reference (GeneBank accession no. MH730557). BLAST (https://blast.ncbi.nlm.nih.gov/Blast.cgi) was used as the first tool in order to screen for similar genomes in the non-redundant nucleotide database. Geneious Prime 2020.2 software bioinformatics platform (https://www.geneious.com) was also used to visualize and in silico search the sequenced genomes, construct the genomic map of NO16 and explore the phylogenetic relationships with the spontaneously induced NO16-like phages from the different strains of *V. anguillarum*. Geneious Tree Builder was used for the phylogenetic tree using the Neighbor-Joining method and Tamura-Nei genetic distance model (1000 bootstraps). Gene prediction for the novel phage genomes was conducted by combining Glimmer 3 (default settings) [1] and Prodigal v2.6.3 (-p meta; procedure: meta) [2]. Genomic annotation was performed both automatically by Rapid Annotation Subsystem Technology (R.A.S.T.) [3] and complemented manually using protein Basic Local Alignment Tool (BLASTP) and Protein Fold Recognition Server, Phyre2 [4] with cutoff e- value: 6E-86 and confidence level > 98%, respectively. Progressive MAUVE algorithm [5] was used in order to align whole genomes and evaluate the synteny of under comparison genomic regions. The novelty of the NO16 and NO16-like phage family was further documented by the construction of a dendrogram in GRAViTy

(http://gravity.cvr.gla.ac.uk, on 9 August 2021) against the database DB-B: Baltimore Group Ib—Prokaryotic and archaeal dsDNA viruses (VMRv34). The illustration of the nwk file was done using the interactive tree of life (iTOL) online software (https://itol.embl.de/) [6].

Text S2: HMM construction. The HMM models were based on a number of different amino acid sequences, which were retrieved by BLASTP. All sequences that were used to feed the HMMs originated from different *Vibrio* species and corresponded to homologous amino acid sequences with query coverage ≥ 95%. Depending on the available GenBank data, which could fulfil the aforementioned criteria, 26 sequences, 15 sequences and 97 sequences were used for building HMMs for DJR MCP, ATPase and SAH proteins, respectively. The construction of HMM was performed by HMMER software v3.3.2 (http://hmmer.org/) using default settings and E-value: 0.01 [7,8]. EBI Jackhammer search tool [1] was also used as a fast evaluation tool to assess HMMs in bacterial genomes prior to the final models, as well as to search against the latest UniProt databases. The final decision for the number of NO16 hits in the *Vibrio* database was taken after combining the results of the models and keeping only the overlapping positive hits for all three. Information about geographic locations and isolation sources was retrieved from the Batch Entrez function of NCBI (https://www.ncbi.nlm.nih.gov/sites/batchentrez).

The *Vibrio* database which was used for the screening contains 8,678 Vibrio genomes, both known and unknown species. This was created by combining the collection of 1,874 previously analyzed genomes [9], extraction of further assembled genomes from GenBank and the assembly of genomes that had only been submitted to the SRA as reads (April 2019). The process for quality control and genome assembly has previously been described (https://github.com/zheminzhou/EToKi). This resulted in a database where genome assembly and gene calling have been also be done in a consistent manner.

TB **Text S3: Bacteriophage dynamics**. Green Premix Ex Taq II (Tli RNase H Plus) (Takara Bio Europe AB, Sweden) was used as master mix along with primers and RNAse- free water following the manufacturer's protocols for 3-step qPCR in order to amplify the targeted genomic area. The qPCR reactions were performed in hard-shelled PCR low profile, semi-skirted plates (BioRad, USA) covered by an adhesive plate sealing film (BioRad, USA) using a Bio-Rad CFX Connect (BioRad, USA) machine.

Text S4: Quantification of phage and bacteria dynamics.

Optical density

One mL was retrieved to measure optical density at 600 nm (OD_{600}) using Novaspec Plus Visible Spectrophotometer (Amersham Biosciences, Uppsala, Sweden).

Bacterial counts

Samples of 0.2 mL were fixed in glutaraldehyde at 2.5% final concentration and stored at -80 °C. They were later prepared for flow cytometry by 100x dilution with Tris-EDTA buffer and stained with 10x diluted SYBR green. Measurements were done on FACS Canto II (BD Biosciences, USA) according to previously described method [10], and calibrated with BD Trucount tubes (BD Biosciences, USA).

Serial dilutions of 20 uL samples from all different conditions and replicates were done in 96-well plates (Cellstar, Greiner Bio-One, Austria) using SM buffer as diluent. Pfus were formed using marine agar containing 6-well plates, on which individual dilutions mixed with bacterial host and soft agar, were poured using same volumes as described above. Pfu counts were noted one day later after overnight incubation of the 6-well plates at room temperature.

Gene expression levels

Based on the phage NO16 genome, the expression levels of specific genes related to prophage biology, biofilm formation and quorum sensing (QS) were assessed under various conditions during both phage integration and prophage induction experiments. Putative transposon-related DNA binding protein (gene 6), S-adenosylhomocysteine hydrolase (gene 7), double-jelly roll capsid protein (gene 19) and putative ATPase (gene 21) were genes of major importance for the life cycle of the phage according to their annotated functions (gene 19).

During all time points of both phage integration and phage induction experiments, two aliquots of 1.5 mL were sampled from all replicates. Stop solution (95% EtOH, 5% phenol) [11] was immediately added at a 0.2-volume ratio and samples were stored in -80 ºC. To extract RNA [12], samples were centrifuged at 10,000 *g* for 15 min at 4 ºC and supernatant was discarded. Pelleted cells were resuspended in 200 μL solution I (0.3 M sucrose , 0.01 M NaOAc, pH 4.5) at 4 ºC and vortexed. Right after, 200 μL solution II (2% SDS, 0.01M NaOAc, pH 4.5) were added while following gentle mixing. Samples were heat treated at 65 °C for 1.5 min and then 400 μL

65 °C phenol:water (pH 4.5-4.7) were added following vortex and incubation at 65 °C for 3 min. The samples were then snap frozen in liquid nitrogen for 15 s and centrifugation at 15,500 *g* for 10 min at room temperature separating RNA from organic phase. After transferring the upper phase to new tubes containing 200 µL 65 °C phenol:water (pH 4.5-4.7) and vortex, the 65 °C incubation, snap freeze and centrifugation steps were repeated. The new upper phase was again transferred to new tubes and RNA was precipitated by adding 50 µL 3 M NaOAc (pH 4.7) and 1 mL 96 % EtOH and vortexed. Samples were stored at -80 °C overnight and the following day RNA was pelleted by centrifuging at 15,500 *g* for 10 min at 4 °C. Supernatant was removed and following two washing steps with 70% EtOH the tubes containing RNA pellet were left to dry for 30 min with open lids. Last, the RNA was resuspended in 50 µL nuclease-free water and immediately saved at –80 °C.

The extracted RNA was treated with DNAse I, following manufacturer's instruction (Roche, Sigma-Aldrich, USA). Deactivation of DNAse I was done by phenol extraction. Briefly, 130 µL nuclease-free water and 200 µL acidic phenol were added to the RNA samples, vortexed and centrifuged at 15,500 g for 5 min. The upper phase was transferred to new tubes and precipitated with 67 µL 3 M Na_2OAc and 500 µL cold 96 % EtOH, vortexed and incubated at -20 ° C for 20 min. Following centrifugation at 10,000 *g* for 10 min, supernatant was discarded and pellet was washed with 70% EtOH. RNA-containing tubes were left to dry at room temperature for 30 min and the dry pellet was resuspended in 50 µL nuclease-free water. RNA concentration and quality control was checked using NanoDrop 2000c spectrophotometer (Thermo Scientific, USA).

Reverse transcription was done with the Thermo Scientific Revert Aid first-strand cDNA synthesis kit, following the manufacturer's instructions using 1000 ng RNA (Thermo Scientific, USA). cDNA was quantified using qPCR as previously described.

During a parallel study using whole-cell spike-in normalization of gene expression data, we became aware that under circumstances similar to this study, normalization to regular internal reference genes, such as *recA*, were inappropriate for comparing gene expression of cells in the exponential growth phase with cells in a stationary growth phase (Mauritzen et al., unpublished). Thus, to find a suitable internal reference gene for the original extensive experiment,

we subsequently tested five candidate genes (selected based on RNA- seq data that showed a stable expression in 2 different wildtype *V. anguillarum* strains at exponential and early stationary growth phase) and benchmarked their stability of expression against the whole-cell spike-in normalization. Based on this, a gene involved in the Cytochrome C complex (PN41_07635) was used to normalize the real time PCR data as it remained the most stably expressed gene at the tested conditions [13, 14].

References

1. Delcher, A.L.; Bratke, K.A.; Powers, E.C.; Salzberg, S.L. Identifying bacterial genes and endosymbiong DNA with Glimmer. *Bioinformatics* **2007**, *23*, 673–679.
2. Hyatt, D.; Chen, G.-L.; LoCascio, P.F.; Land, M.L.; Larimer, F.W.; Hauser, L. Prodigal: Prokaryotic gene recognition and translation initiation site identification. *BMC Bioinform.* **2010**, *11*, 119.
3. Overbeek, R.; Olson, R.; Pusch, G.D.; Olsen, G.J.; Davis, J.J.; Disz, T.; Edwards, R.A.; Gerdes, S.; Parrello, B.; Shukla, M.; et al. The SEED and the rapid annotation of microbial genomes using subsystems technology (RAST). *Nucleic Acids Res.* **2014**, *42*, 206–214.
4. Kelly, L.A.; Mezulis, S.; Yates, C.; Wass, M.; Sternberg, M. The Phyre2 web portal for protein modelling, prediction, and analysis. *Nat. Protoc.* **2015**, *10*, 845–858.
5. Darling, A.C.E.; Mau, B.; Blattner, F.R.; Perna, N.T. Mauve: Multiple Alignment of Conserved Genomic Sequence with Rearrangements. *Genome Res.* **2004**, *14*, 1394–1403.
6. Letunic, I.; Bork, P. Interactive Tree of Life (iTOL) v4: Recent updates and new developments. *Nucleic Acids Res.* **2019**, *47*, 256–259.
7. Mistry, J.; Finn, R.D.; Eddy, S.R.; Bateman, A.; Punta, M. Challenges in homology search: HMMER3 and convergent evolution of coiled-coil regions. *Nucleic Acids Res.* **2013**, *41*, e121–e121.
8. Finn, R.D.; Clements, J.; Arndt, W.; Miller, B.L.; Wheeler, T.J.; Schreiber, F.; Bateman, A.; Eddy, S.R. HMMER web server: 2015 Update. *Nucleic Acids Res.* **2015**, *43*, W30–W38.
9. Castillo, D.; Kauffman, K.; Hussain, F.; Kalatzis, P.; Rørbo, N.; Polz, M.F.; Middelboe, M. Widespread distribution of prophage-encoded virulence factors in marine Vibrio communities. *Sci. Rep.* **2018**, *8*, 9973.
10. Castillo-Hair, S.M.; Sexton, J.T.; Landry, B.P.; Olson, E.J.; Igoshin, O.A.; Tabor, J.J. FlowCal: A User-Friendly, Open Source Software Tool for Automatically Converting Flow Cytometry Data from Arbitrary to Calibrated Units. *ACS Synth. Biol.* **2016**, *5*, 774–780.
11. Bernstein, J.A.; Khodursky, A.B.; Lin, P.H.; Lin-Chao, S.; Cohen, S.N. Global analysis of mRNA decay and abundance in *Escherichia coli* at single-gene resolution using two-color fluorescent DNA microarrays. *Proc. Natl. Acad. Sci. USA* **2002**, *99*, 9697–9702.
12. Gummesson, B.; Shah, S.A.; Borum, A.S.; Fessler, M. Valine-Induced Isoleucine Starvation in Escherichia coli K-12 Studied by Spike-In Normalized RNA Sequencing. *Front. Genet.* **2020**, *11*, 144.
13. Tan, D.; Lo Svenningsen, S.; Middelboe, M. Quorum sensing determines the choice of antiphage defense strategy in *Vibrio anguillarum*. *mBio* **2015**, *6*, e00627–15.
14. Ma, Y.-J.; Sun, X.-H.; Xu, X.-Y.; Zhao, Y.; Pan, Y.-J.; Hwang, C.-A.; Wu, V.C.H. Investigation of reference genes in vibrio parahaemolyticus for gene expression analysis using quantitative RT-PCR. *PLoS One* **2015**, *10*, e0144362.

II. Supplementary figures

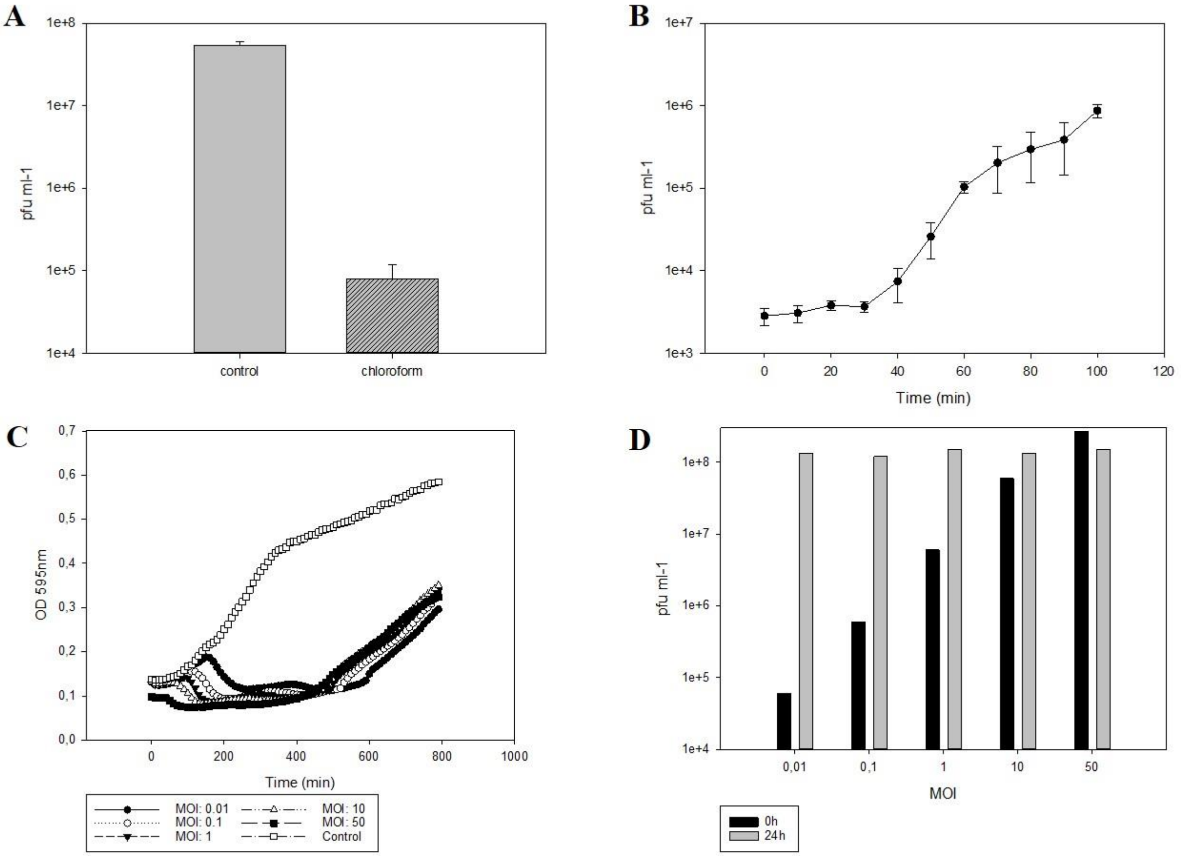

Figure S1. A. Titer comparison of chloroform-treated NO16 phages compared to untreated controls, B. One-step growth curve of phage NO16 over a period of 100min; latency time, 30min and burst size, 31 virions per cell, C. In vitro cell lysis of *V. anguillarum* strain A023 by NO16 phage under a wide range of MOI; 0.01, 0.1, 1, 10 and 50, D. Assessment of free NO16 phages after 24h infection under a wide range of MOI; 0.01, 0.1, 1, 10 and 50. The values are means ±standard deviation of the three replicates.

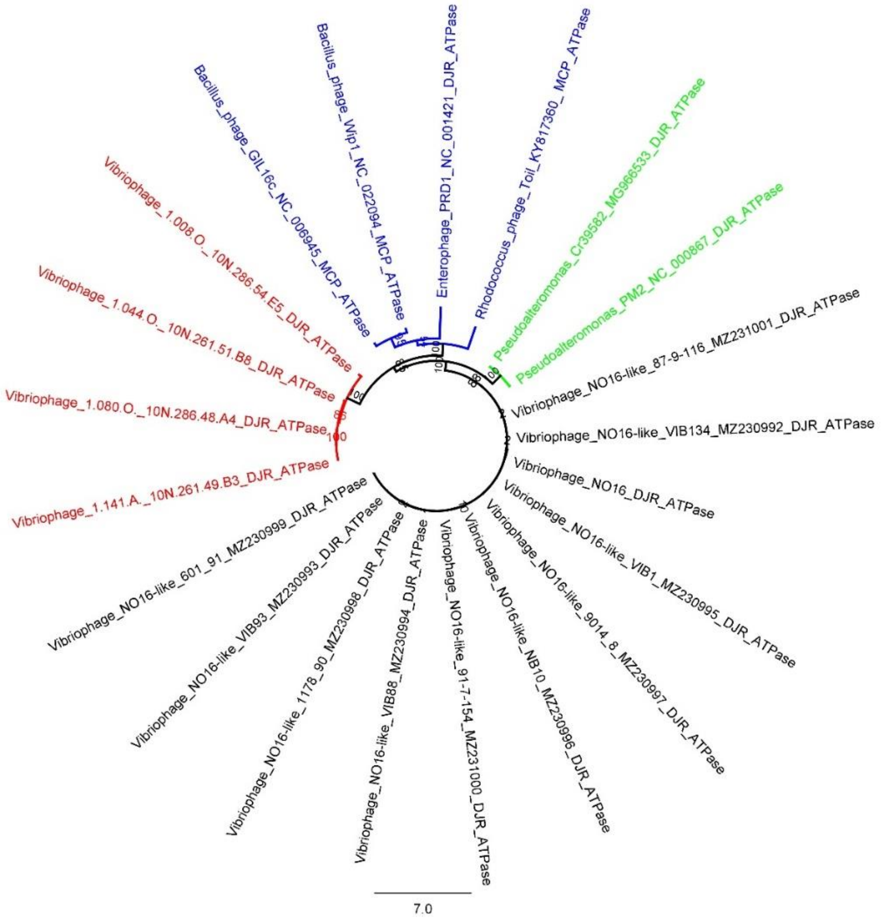

Figure S2.

ML phylogenetic tree (RAxML) based on the concatenated major capsid protein and ATPase amino acid sequences. The phage families of *Tectiliviricetes* form distinct monophyletic taxa supported by high level of significance (100%): Autolykiviridae in red, Tectiviridae in blue, Corticoviridae in orange and NO16 family phages in black font.

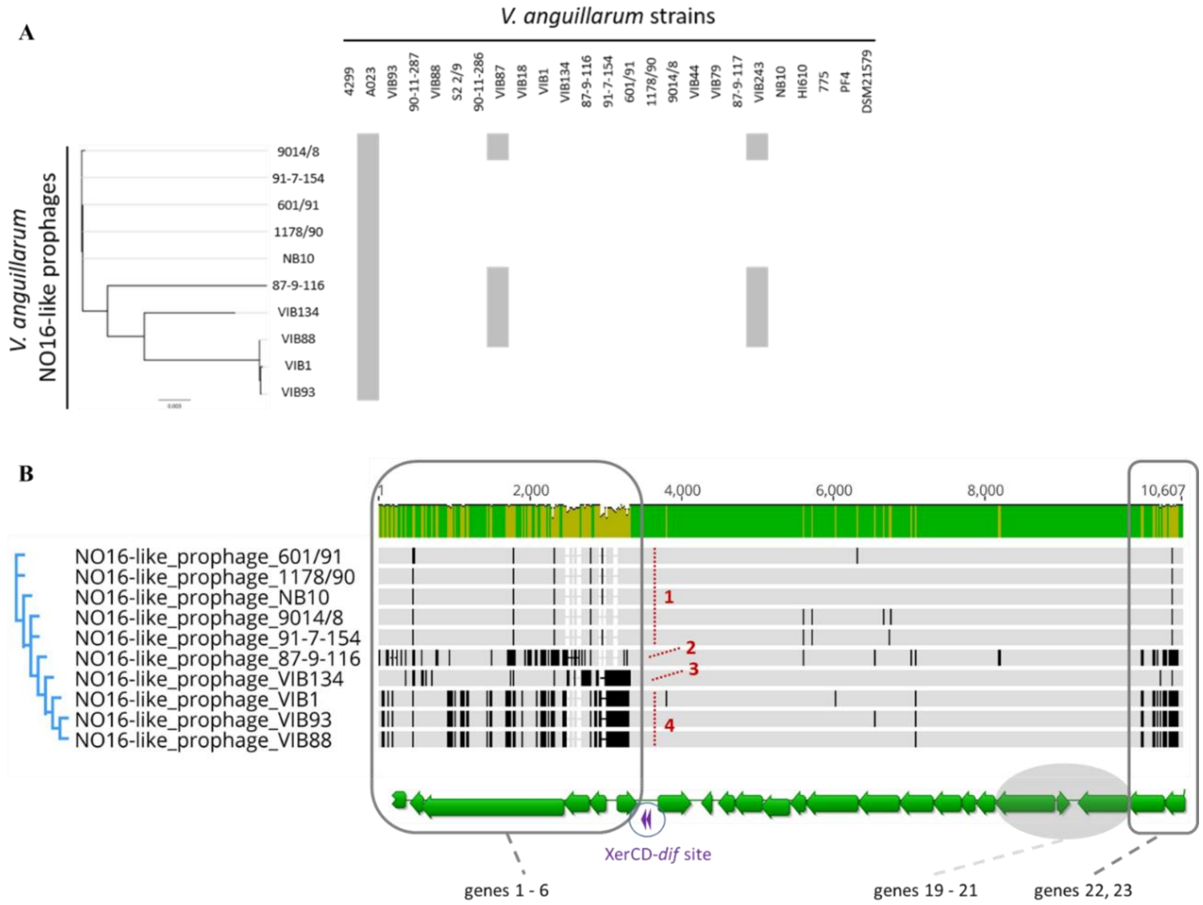

Figure S3. A. Host range analysis of the 10 spontaneously induced NO16 phages against 25 different *V. anguillarum* strains. Bacterial growth inhibition is noted with a shaded square. Phylogenetic relationships of the phages are illustrated by a Neighbor-Joining tree of 1000 bootstraps, B. Genomic alignment of the sequenced NO16 10 kb-phages. Boxes indicate the genomic areas where most SNPs were recorded, leading to the formation of 4 closely related subgroups.

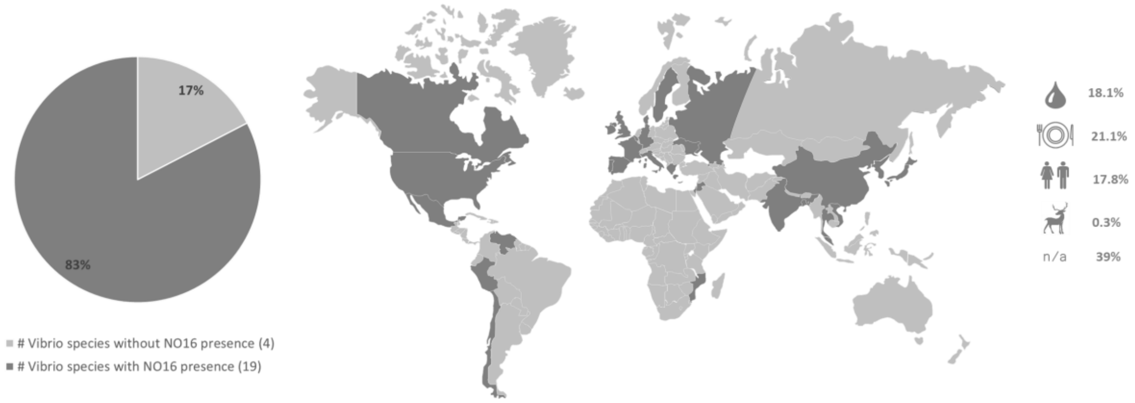

Figure S4. *Geographical prevalence and isolation sources of NO16-like prophages in the Vibrio database. The 97% of the sequences in the Vibrio database is represented by 23 Vibrio species other than V. anguillarum. One of the 23 Vibrio clusters contains the unidentified Vibrio sp. where NO16 is also present. Out of the 23 most prevalent Vibrio species, 19 carry the N016 prophages (V. parahaemolyticus, V. cholerae, V. vulnificus, V. lentus, unclassified Vibrio sp., V. splendidus, V. alginolyticus, V. coralliilyticus, V. cyclitrophicus, V. harveyi, V. crassostreae, V. metoecus, V. campbellii, V. mimicus, V. diabolicus, V. tasmaniensis, V. jasicida, V. mediterranei, V. owensii), whereas only four of them do not (V. breoganii, V. fluvialis, V. nigripulchritudo, V. diazotrophicus).*

XerCD - *dif* site

	In silico V. anguillarum contigs (XerC … XerD)	Sequenced NO16 phages (XerC … XerD)
NO16-like prophage 91-7-154	GGTGCGCATTATG-TATA-ATGTGGTAGCC	GGTGCGCATTATG-TATA-ATGTGGTAAAC
NO16-like prophage 601/91	GGTGCGCATTATG-TATA-ATGTGGTAGCC	GGTGCGCATTATG-TATA-ATGTGGTAAAC
NO16-like prophage 1178/90	GGTGCGCATTATG-TATA-ATGTGGTAGCC	GGTGCGCATTATG-TATA-ATGTGGTAAAC
NO16-like prophage 9014/8	GGTGCGCATTATG-TATA-ATGTGGCAGCC	GGTGCGCATTATG-TATA-ATGTGGTAAAC
NO16-like prophage NB10	GGTGCGCATTATG-TATA-ATGTGGTAGCC	GGTGCGCATTATG-TATA-ATGTGGTAAAC
NO16-like prophage 87-9-116	GGTGCGCATTATG-TATA-ATGTGGTAGCC	GGTGCGCATTATG-TATA-ATGTGGTAAAC
NO16-like prophage VIB1	GGTGCGCATTATG-TATA-ATGTGGCAGCC	GGTGCGCATTATG-TATA-ATGTGGTAAGT
NO16-like prophage VIB88	GGTGCGCATTATG-TATA-ATGTGGCAGCC	GGTGCGCATTATG-TATA-ATGTGGTAAGT
NO16-like prophage VIB93	GGTGCGCATTATG-TATA-ATGTGGCAGCC	GGTGCGCATTATG-TATA-ATGTGGTAAGT
NO16-like prophage VIB134	GGTGCGCATTATG-TATA-ATGTGGTAGCC	GGTGCGTATTATA-TATC-ATGTGGTAAGT
NO16 phage	GGTGCGCATTATG-TATA-ATGTGGTAGCC	GGTGCGCATTATG-TATA-ATGTGGTAAAC
dif 1 V. cholerae	AGTGCGCATTATG-TATG-TTATGTTAAAT	
V. anguillarum attB1 – VAIϕ	AGTGCGCATTATG-TATG-TTATGTTAAAT	
V. anguillarum attB2 – VAIϕ	AGTGCGCATTATG-TATA-TTATGTTAAAT	

Figure S5. The 28-bp dif sites which facilitate the NO16 integration in 10 different *V. anguillarum* strains that carry the prophage. On the left column are the dif sites that were identified in silico from the resubmitted original contigs of the *V. anguillarum* collection (35) while their counterparts in the integration process of VAI phage in *V. anguillarum* and CTX phage in *V. cholerae* are as well included. On the right column are the dif sites that were identified in the spontaneously induced and sequenced NO16 phages.

III. Supplementary Tables (includes also 2 additional excel files)

Supplementary Table S1: *V. anguillarum* strains that were used to assess the lytic spectrum of the novel phage NO16.

**Strains**	**Serotype**	**Genome accesion number**
**4299**	**O2b**	**CP011458/CP011459**
**VIB64**	**O1**	**CP010036/CP010037**
**VIB93**	**O1**	**CP011438/CP011439**
**90-11-287**	**O1**	**CP011475/CP011476**
**VIB88**	**O1**	**CP010042/CP010043**
**S2 2/9**	**O1**	**CP011472/CP011473**
**90-11-286**	**O1**	**CP011460/CP011461**
**VIB87**	**O1/VaNT1**	**CP010040/CP010041**
**VIB18**	**O1**	**CP011436/CP011437**
**VIB1**	**O1**	**CP010291/CP010292**
**VIB134**	**O1**	**CP010034/CP010035**
**87-9-116**	**O1**	**CP010044/CP010045**
**91-7-154**	**O1**	**CP010082/CP010083**
**601/91**	**O1**	**CP010076/CP010077**
**178/90**	**O1**	**CP011470/CP011471**
**9014/8**	**O1**	**CP010038/CP010039**
**VIB44**	**O1**	**CP010032/CP010033**
**VIB79**	**O1**	**CP011468/CP011469**
**87-9-117**	**O1**	**CP010046/ CP010047**
**NB10**	**O1**	**LK021130/LK021129**

Supplementary Table S2: List of the features of primers that were used for quantitative PCR reactions of this study in order to assess integration site DNA copies and expression levels of selected genes in various experimental condition.

Target	Sequence	Name	Length (bp)	Product size (bp)	GC%	Tm (ºC)
Phage biology related genes						
Integration site	GGGATTTGAAGCGAGGGAGT	F 2r	20	142	55	59.7
	GCTACATCCCTAGAAAGCGC	R 1r			55	58.5
Putative transposon-related DNA binding protein	GGCGAAAAGGCTACTGCTCT	F trn	20	152	55	60.4
	ACGCTCAGCAATCTCATGGA	R trn			50	59.5
S-adenosylhomocysteine hydrolase	ACGTAAAGCCCGTTACCGTT	F SahH	20	120	50	60
	CAGTCCAGCGCGAATCATTG	R SahH			55	60
double-jelly roll capsid protein	CGGTCACGTCTGCTGAGTAG	F DJR2	20	104	60	60.2
	CAAGCACTCAAGCGCAGTTT	R DJR2			50	60
putative ATPase	CGAGCGCCAAGAACTCAAAG	F ATPase	20	111	55	59.8
	AGGCGTCACAGGTCATTGAG	R ATPase			55	60
Quorum sensing related genes						
mta/sah	CTGGTTGACCTGCCATTTGC	F mta/sah	20	128	55	60
	CGGTTCTGCAGGTGGTTTTG	R mta/sah			55	60
Biofilm related genes						
vpsL	AGTGACTCCTTTTGGTGCGT	F vpsL	20	194	50	59.8
	GTGCCCAGCCAGTAATTCCT	R vpsL			55	60
vpsM	ACTCATGAACAGCGTGGTGA	F vpsM	20	104	50	59.6
	GCGTCTTCACCACCAAAACC	R vpsM			50	59.6
vpsN	ATTGCGGGTGGATTCACTGA	F vpsN	20	124	50	59.7
	TGAGAATGTCACCAGGCTGA	R vpsN			50	58.6
vpsO	GCAGGAGTCGTGACAGACAA	F vpsO	20	169	55	60
	CGAGTATGGTCGAGTCCACG	R vpsO			60	60
Candidate calibrator genes						
Cytochrome C	AGCCCATTTGGTCGAAGAGG	F 07365	20	121	55	60
	CAATACTTTCAGCGGCGTCG	R 07365			55	60
Hypothetical protein	AAAATCGGTGCGGGTACTGT	F 15920	20	132	50	60
	CGCCACTACGCTCAATGAGA	R 15920			55	60.2
Osmotically inducible protein OsmC	TGGTCATTCGGTGGTGATGG	F 16225	20	138	55	60
	GCAGTGACCTGTTGATTGGC	R 16225			55	59.8
Hypothetical protein	AGGGAATGGCTCTCACTTGC	F 13180	20	115	55	60
	TTAGCCGCGGAACTGTTTCT	R 13180			50	60
recA	CTTTTGGTGTCAGTGCAGCC	F RecA	20	104	55	60
	ACCTGATACGGGCGAACAAG	R RecA			55	60.1
gyrB	TTCTCACCCATTGCCGACTC	F gyrB	20	108	55	60

	CGCGGTGGTTTCAGTGAAAG	R gyrB			55	60
rpoS	GGATAGTGCGCGTTTGGTTC	F rpoS	20	120	55	59.9
	GCTTGATCCGTGCGGTAGAA	R rpoS			55	60.5
dnaK	AAGCTCAGTAATGGCCGCTT	F dnaK	20	102	50	60
	GCGGACCAAATGATTCACGG	R dnaK			55	59.9

Table legends for Supplementary excel files:

Supplementary Table S3: Similarity of NO16 phage genomic content on both the nucleotide level (BLASTn) and the protein fold level (phyre2), against the available database. Additional comparisons on the aminoacid/protein level (Jackhammer, BLASTP) were done for the DJR MCP and ATPase proteins which constitute products of the core genes of non-tailed bacteriophages.

Supplementary Table S4: Individual and overlapping hits of the HMMs built for DJR MCP, ATPase and SAH hydrolase proteins against the *Vibrio* genomes database. Reference sequences as well as explanatory information for each species mentioned in Fig. 4 are extensively reported.
